# 2492 Risk of readmission after discharge from skilled nursing facilities following heart failure hospitalization

**DOI:** 10.1017/cts.2018.303

**Published:** 2018-11-21

**Authors:** Himali Weerahandi, Li Li, Jeph Herrin, Kumar Dharmarajan, Lucy Kim, Joseph Ross, Simon Jones, Leora Horwitz

**Affiliations:** 1 NYU Lutheran, Brooklyn, NY, USA; 2 Yale School of Medicine, New Haven, CT, USA; 3 NYU School of Medicine, New York, NY, USA; 4 Yale University School of Medicine, New Haven, CT, USA; 5 School of Medicine, New York University, New York, NY, USA

## Abstract

OBJECTIVES/SPECIFIC AIMS: Determine timing of risk of readmissions within 30 days among patients first discharged to a skilled nursing facilities (SNF) after heart failure hospitalization and subsequently discharged home. METHODS/STUDY POPULATION: This was a retrospective cohort study of patients with SNF stays of 30 days or less following discharge from a heart failure hospitalization. Patients were followed for 30 days following discharge from SNF. We categorized patients based on SNF length of stay (LOS): 1–6 days, 7–13 days, 14–30 days. We then fit a piecewise exponential Bayesian model with the outcome as time to readmission after discharge from SNF for each group. Our event of interest was unplanned readmission; death and planned readmissions were considered as competing risks. Our model examined 2 different time intervals following discharge from SNF: 0–3 days post SNF discharge and 4–30 days post SNF discharge. We reported the hazard rate (credible interval) of readmission for each time interval. We examined all Medicare fee-for-service (FFS) patients 65 and older admitted from July 2012 to June 2015 with a principal discharge diagnosis of HF, based on methods adopted by the Centers for Medicare and Medicaid Services (CMS) for hospital quality measurement. RESULTS/ANTICIPATED RESULTS: Our study included 67,585 HF hospitalizations discharged to SNF and subsequently discharged home [median age, 84 years (IQR; 78–89); female, 61.0%]; 13,257 (19.2%) were discharged with home care, 54,328 (80.4%) without. Median length of SNF admission was 17 days (IQR; 11–22). In total, 16,333 (24.2%) SNF discharges to home were readmitted within 30 days of SNF discharge; median time to readmission was 9 days (IQR; 3–18). The hazard rate of readmission for each group was significantly increased on days 0–3 after discharge from SNF compared with days 4–30 after discharge from SNF. In addition, the hazard rate of readmission during the first 0–3 days after discharge from SNF decreased as the LOS in SNF increased. DISCUSSION/SIGNIFICANCE OF IMPACT: The hazard rate of readmission after SNF discharge following heart failure hospitalization is highest during the first 6 days home. Length of stay at SNF also has an effect on risk of readmission immediately after discharge from SNF; patients with a longer length of stay in SNF were less likely to be readmitted in the first 3 days after discharge from SNF.

